# Enhanced bioavailability of a krill oil-based milk thistle extract formulation: *in vitro* and human studies

**DOI:** 10.29219/fnr.v70.13256

**Published:** 2026-01-07

**Authors:** Karin Engelhart-Jentzsch, Ann-Kathrin Gantenbein, Christiane Schön, Manfred Wilhelm, Lena Stadelmayer, Lisa Pross, Tatjana Kaiser-Zimmermann, Gregorio Guerrero, Benjamin Assad Jaghutriz, Claudia Reule

**Affiliations:** 1 BioTeSys GmbH, Esslingen, Germany; 2 Department of Mathematics, Natural and Economic Sciences, Ulm University of Applied Sciences, Ulm, Germany; 3 Wörwag Pharma GmbH & Co.KG, Böblingen, Germany

**Keywords:** silymarin, silybin, silibinin, milk thistle, krill oil, pharmacokinetic, MASLD, Caco-2 model, in vitro, human study, phospholipids, biofactor

## Abstract

**Background/Objectives:**

The milk thistle plant (*Silybum marianum*) is known for its hepatoprotective properties. However, the poor water solubility of silymarin limits its dissolution in the intestinal tract and restricts its bioavailability following oral administration.

**Methods:**

To improve bioavailability, special formulations, in particular micellar solubilization, are explored. In this study, we examined the transport rate of silymarin in a krill oil-based formulation across a Caco-2 epithelial barrier after upstream digestion simulation *in vitro.* Furthermore, in a randomized cross-over design study the bioavailability of the krill oil-based formulation was investigated after single dose intake in fasting conditions in healthy participants.

**Results:**

We could demonstrate that the apparent transport coefficient of silybin, measured as lead substance of milk thistle extract, across the epithelium is efficiently boosted by a krill oil formulation, resulting in a 28% increase compared to silymarin powder. Consistent with these findings, a significant enhancement of bioavailability (*P* < 0.0001) was demonstrated for the krill oil-based formulation in comparison to the milk thistle extract resulting in an 8.59-fold higher AUC_0-8h_ and a 15.08-fold greater C_max_ of silybin and faster uptake kinetic after single dose intake.

**Discussion and Conclusions:**

These findings suggest that phospholipid-based delivery systems offer a promising strategy for improving the efficacy of lipophilic bioactives. Furthermore, the combination of krill oil with milk thistle extracts efficiently provides silybin, PUFAs, and choline, which are important nutrients contributing to liver and heart health.

## Popular scientific summary

- Milk thistle extract is known for its hepatoprotective features; however, the poor water solubility limits its oral bioavailability.
- A combination of milk thistle extract with krill oil (rich in phospholipids) showed a superior transport rate of silymarin across a Caco-2 epithelial barrier as well as a significantly higher oral bioavailability in healthy participants.
- This study demonstrates that phospholipid-based delivery systems are a promising strategy for improving the intestinal uptake of lipophilic bioactives.

Metabolic dysfunction-associated steatotic liver disease (MASLD), with an estimated global prevalence of 38%, has emerged as a leading cause of chronic liver disease worldwide (1). The pathophysiology of MASLD is intricately linked to a cluster of cardiometabolic risk factors, including insulin resistance, type 2 diabetes, and dyslipidemia – often characterized by hypertriglyceridemia and an unfavorable lipoprotein profile (2). Consequently, nutritional strategies aimed at mitigating the development and progression of MASLD are of significant scientific and clinical interest.

Extracts from the milk thistle plant (*Silybum marianum*) have long been investigated for their hepatoprotective properties. The seeds contain a complex of flavonolignans known as silymarin (3), which is recognized for its potent antioxidative, anti-inflammatory, antifibrotic, and regenerative effects on the liver (4, 5). However, the health benefits of silymarin are substantially limited by the poor oral bioavailability of its primary and most active component, silybin (also called silibinin and used as synonym in scientific literature) (6). This limitation is a direct consequence of silybin’s low aqueous solubility, which impairs its dissolution and subsequent absorption within the intestinal tract (7).

To overcome this pharmacokinetic challenge, advanced formulation technologies are often employed. There are numerous approaches that have been studied to enhance the bioavailability of poorly soluble substances, such as several nanotechnologies (8), synthetic emulsifiers (9) and micellular solubilization (10). In addition, other natural bioenhancers such as lysergol, piperine and fulvic acid have been used to increase the bioavailability of silymarin (11).

This study investigates a novel approach that combines two natural food-grade ingredients: milk thistle extract and krill oil. The rationale for this formulation is twofold. Firstly, krill oil is a rich source of phospholipids (12), which act as natural emulsifiers capable of forming micelles that can enhance the solubilization and absorption of lipophilic compounds (13) such as silybin, thus potentially eliminating the need for synthetic excipients (14). Secondly, this combination creates a formulation of complementary bioactives, uniting silybin with the omega-3 polyunsaturated fatty acids (PUFAs) and choline from krill oil, all of which are independently recognized for contributing to hepatic and cardiovascular health (4, 5, 15, 16).

While the systemic absorption of eicosapentaenoic acid (EPA) and docosahexaenoic acid (DHA) from krill oil is well-documented (14, 17, 18), the primary objective of this investigation was to determine whether this phospholipid-rich matrix could significantly enhance the bioavailability of silybin. To address this, a sequential, two-step experimental design was implemented. In the first step, we assessed the transepithelial transport of silybin in an *in vitro* Caco-2 cell monolayer model, a well-established system that mimics the human intestinal epithelial barrier (19). Acknowledging the inherent limitations of extrapolating *in vitro* findings to *in vivo* outcomes setting (20, 21), the second step of the experiments consisted of conducting a clinical study with healthy human participants. The aim of the clinical study was to measure and compare the bioavailability of silybin from the novel milk thistle extract and krill oil in combination versus a standard pure milk thistle extract alone.

## Materials and methods

### In vitro study

#### Chemicals and cell line

Mucin, trypsin, pepsin, bile extract, pancreatin, methylthiazolyldiphenyl-tetrazolium bromide (MTT) and all other chemicals were purchased from Merck Sigma-Aldrich (Darmstadt, Germany). Minimal essential medium (MEM), fetal bovine serum (FBS), non-essential amino acids (NEA), penicillin/streptomycin and trypsin/ethylenediaminetetraacetic acid (EDTA) solution were purchased from PAN Biotech (Aidenbach, Germany). Caco-2 cells (ATCC® HTB-37) were purchased from the American Type Culture Collection (Manassas, USA).

Silymarin was obtained as milk thistle extract powder (80% silymarin) from Gonmisol Fine Ingredients (Barcelona, Spain), and krill oil as Krill Oil (Superba Boost®) from Aker BioMarine (Lysaker, Norway).

#### Silymarin in krill oil formulations

Krill oil formulations containing three different silymarin concentrations were prepared. All three formulations contained 1.2 g Krill Oil (Superba Boost®) and in addition 150 mg (Mix A), 80 mg (Mix B) or 40 mg (Mix C) milk thistle extract powder (80% silymarin) were added. A total of 40 mg of milk thistle extract powder (80% silymarin) without krill oil served as control. The concentrations were selected in orientation to later acceptable doses in food supplements to be used in different countries.

Silymarin in krill oil formulations were provided by Wörwag Pharma (Böblingen, Germany).

#### Cell culture

Caco-2 cell line was cultured as described by the provider in MEM supplemented with 20% FBS, 1% NEAs, 100 U/mL penicillin, and 100 µg/mL streptomycin. Cells were maintained in a cell culture incubator under normal conditions (37°C, 5% CO_2_). Cells were seeded at a density of 1 × 10^4^ cells/cm^2^. Cell culture medium was changed twice a week.

In order to show the impact of the combination krill oil and milk thistle extract on the transepithelial transport of silybin across a Caco epithelium *in vitro*, a constant krill oil concentration was used while varying the milk thistle extract concentrations. Prior to the cell experiment, a simulation of the gastro-intestinal processes, including enzymatic and pH changes, was performed to account for potential metabolic changes.

#### In vitro digest simulation

Since it is known that physicochemical properties of specific bioactive classes such as polyphenols, are altered during gastrointestinal passage and that this biotransformation influences the bioavailability (22), the silymarin in krill oil formulations as well as the milk thistle extract powder underwent a simulation of the gastrointestinal tract passage before the transepithelial transport experiments. Potential metabolic changes of silymarin or the formation of micelle structures may occur, making the resulting solution more representative of *in vivo* conditions.

The *in vitro* digest procedure was performed according to Kraft (23) with minor deviations. In detail, the silymarin in krill oil formulations were added in the respective intended single dose concentration (150/80/40 mg silymarin in 1.2 g krill oil) to 120 mL of an aqueous mucin/pepsin solution (pH 2). As control, 40 mg milk thistle extract powder was mixed with 120 mL of an aqueous mucin/pepsin solution (pH 2). All solutions were incubated for 2 h at 37°C under constant agitation. Thereafter, pancreatin, trypsin, and bile extract were added, and the pH was adjusted to pH 7.5. The solutions were incubated for a further 4 h at 37°C under constant agitation.

A digestion solution without any added test item served as vehicle control for dose finding and transport experiments.

#### Dose finding

Dose findings were performed for Mix A, B, and C as well as for the control. Caco-2 cells were supplemented with seven concentrations of the respective solution obtained from the *in vitro* digest simulation in a concentration range of 0.2–20%. Maximal applicable dose was determined by means of cell viability using MTT conversion assay.

#### Transepithelial transport of silybin

The transepithelial transport of silybin was assessed using Caco-2 models as described earlier (24). Briefly, the cells were seeded into 6-well inserts (ThinCertTM with 3 μm pore size, Greiner Bio-one, Germany) and cultured over 14–21 days to build a differentiated epithelium. The degree of differentiation was checked regularly by means of measurements of transepithelial electrical resistance (TEER) using an epithelial volt/ohm meter (EVOM; WPI, USA).

For all groups, six differentiated Caco-2 models were supplemented at the apical side with 2% of the respective digest solution in medium for 24 h. As control, three models were supplemented with control digest solution. At the end of the incubation period, medium samples of the basal compartment were withdrawn and analyzed for their silybin content.

#### Analytics

The content analysis of silybin in media samples was carried out by liquid–liquid extraction with methyl tert-butyl ether (MTBE) followed by Liquid Chromatography-Tandem Mass Spectrometry (LC-MS/MS). In brief, 100 µL medium sample was mixed with 50 µL methanol and 50 µL of internal standard (400 ng/mL naringenin in methanol). After thoroughly mixing the sample, 1 mL of MTBE was added. The sample was mixed on a Vortex and further treated in an ultrasonic bath at 24°C. The samples were centrifuged for 3.5 min at 13,200 rpm and the upper organic layer was collected. After drying under nitrogen flow, the samples were resolubilized in 200 µL 50% methanol in water, again centrifuged at 13,200 rpm for 3.5 min and the supernatant transferred to a glass semi micro injection vial. A matrix adjusted calibration curve and control samples were accomplished by the same procedure as above. The range of the calibration curve was 5 to 1,000 ng/mL. Silybin content was analyzed by LC-MS/MS using a Waters XEVO TQ-S micro mass spectrometer coupled to an Acquity H-Class Ultra Performance Liquid Chromatography (UPLC) system. Further detailed LC-MS/MS methodology is provided in the Supplementary Materials (Technical Annex).

#### Statistics

Data of the cell culture study are indicated as mean values ± standard deviation (SD) and the experiments were performed in *n* = 6 for each group. Statistical evaluation was performed using GraphPad Prism version 5.04, (GraphPad Software, Inc.) for calculation of one-way analysis of variance (ANOVA) and Dunnett’s multiple comparisons post-test. *P*-values <0.05 are considered statistically significant.

#### Calculation of the apparent permeability coefficient

For comparison of the transport rate of silybin in the different groups, the respective apparent permeability coefficient (P_app_) was calculated (25).


$$\mathrm{Papp}=\left(\frac{\mathrm{VA}}{\mathrm{area}*\mathrm{time}})*(\frac{[\mathrm{silybin}]\mathrm{acceptor}}{[\mathrm{silybin}]\mathrm{initial}\;\mathrm{donor}}\right)$$

In the apical compartment, the volume (VA) was 2.0 mL, area of the porous membrane surface (area) was 4.52 cm^2^, and the incubation time (time) 86,400 s. Concentrations of silybin were used as measured by LC-MS/MS whereas “acceptor” represents the basolateral part and “initial donor” the apical compartment.

### In vivo study

#### Study design

The *in vivo* study was performed as a randomized, two-way crossover study with a 14 days wash-out phase between assessments. The study visits took place between January 2025 and March 2025 at BioTeSys GmbH, Esslingen, Germany. Participants were recruited using flyers, public notices and the internal database of participants at BioTeSys GmbH. A total of 18 participants completed the study. Eligible persons were healthy, non-smoking adults between 18 and 60 years of age and a Body Mass Index (BMI) of 19 to 30 kg/m^2^. Main exclusion criteria consisted of regular intake of milk thistle, krill oil or fish oil and other supplements possibly interfering with the study within 2 weeks before study start or during study and regular intake of substances affecting blood coagulation. Also, hemoglobin levels of <11 g/dL for women and <12 g/dL for men and liver enzymes (aspartate aminotransferase or alanine aminotransferase) more than 3-fold upper limit resulted in exclusion. A detailed list of the complete inclusion and exclusion criteria is provided in a supplementary file. This study was approved by the Institutional Review Board (IRB) of Landesärztekammer Baden-Württemberg (Approval Number: F-2024-115, Stuttgart, Germany). It was performed in accordance with the guidelines for Good Clinical Practice (GCP) established by the International Council for Harmonisation of Technical Requirements for Pharmaceuticals for Human Use (ICH) and in conformity with the Declaration of Helsinki regarding the treatment of human participants. The study is registered at DRKS (DRKS00035555). Informed consent was obtained from all subjects involved in the study.

#### Composition of study products

The study product Lagosa Triplex® (further described as MTKO) combined milk thistle extract with Krill Oil (Superba Boost®, Aker BioMarine ASA, Lysaker, Norway), refined sunflower oil and L-choline bitartrate (Jinan Jiuan Ester Chemical Co., LTD., Shadong, China) in a soft gelatine capsule (HC Clover Productos y Servicios, S.L., Madrid, Spain). As reference product, pure milk thistle extract was utilized, encapsulated in a hydroxypropyl methylcellulose (HPMC) shell (Capsugel Belgium NV, Bornem, Belgium). Both products contained 50 mg milk thistle extract per capsule (standardized to 80% silymarin). One single dose was equivalent to two capsules (100 mg milk thistle extract). Both study products were provided by Wörwag Pharma (Böblingen, Germany). Participants were randomly assigned to two sequence groups, which were balanced between periods and stratified for sex. The randomization list was generated with the software DatInf RandList V1.5 (Datinf GmbH, Tübingen, Germany).

#### Sampling and data collection

Participants arrived at the study site in the morning following at least a 10-h overnight fast. After measurement of vital signs and pregnancy testing for women of child-bearing potential, participants were laid a permanent venous catheter, followed by a first blood sampling in order to assess safety parameters and baseline values of silybin. Thereafter, the participants were administered two capsules of either milk thistle extract combined with krill oil or the powdered, pure milk thistle extract, respectively, with 250 ml of water. Further blood samplings were drawn 0.5, 1, 1.5, 2, 2.5, 3, 4, 6 and 8 h after intake of the study product. Participants remained in a fasting state until 4 h after study product administration. They followed a standardized meal protocol beginning with the last meal (farmhouse bread with cream cheese and cucumber or tomato) in the evening before the study days. During kinetic days, all participants received standardized breakfast (pretzel with butter, boiled egg and orange juice) 4 h and lunch (spaghetti with tomato sauce, parmesan and fruit yoghurt) 6 h after study product intake. Water was allowed as desired except for 1 h before and 3 h after the study product administration. During this period, water consumption was standardized. Besides the water for the study product intake, 100 mL of water was served after 1 and 2 h post.

#### Sample analysis

Safety blood routine parameters were analyzed at the accredited laboratory SYNLAB Medizinisches Versorgungszentrum, Leinfelden-Echterdingen, Germany.

Silybin content in plasma was determined at BioTeSys GmbH, Esslingen, Germany with the same plant as described above. Blood samples were collected in monovettes containing anticoagulants (EDTA). They were centrifuged at 3,000 x g for 10 min at 4°C latest 60 min after sampling. Appropriate plasma aliquots were stored at −70°C until analysis. For analysis, 200 μL plasma was mixed with 300 μL incubation buffer. After adding 20 μL β-glucuronidase from Helix pomatia, the vials were placed on a shaker for 1 h at 37°C. After cooling to room temperature, 450 μL of 10% formic acid in water, 100 μL of methanol was added before silybin was extracted with 1 mL MTBE via treatment on a vortex mixer and centrifugation. A total of 750 µL of the supernatant were dried down with a nitrogen steam at 40°C in the dark, the residue was solubilized with 150 µL methanol, mixed and centrifuged. 2 µL of the clear supernatant were injected into the LC-MS/MS system.

For quantification purposes a matrix adjusted calibration strategy was used with a calibration range of 1.0 – 400 ng/mL. Interassay precision was 6.3% while the limit of detection (LOD) and limit of quantification (LOQ) were determined to be <0.3 ng/mL (3 x ratio s/n) and < 1 ng/mL (10 x ratio s/n), respectively.

#### Statistics

A sample size of 18 participants (*n* = 18) was selected based on conventional bioavailability studies (26).

As silybin could not be detected in the baseline samples, pharmacokinetic endpoints area under the curve (AUC_0-8h)_, maximum increase in plasma concentration after administration (C_max_) and time to reach C_max_ (T_max_) of silybin were determined by applying the trapezoidal rule with the y-axis (concentration value) and the x-axis (sampling timepoint). AUC and C_max_ were evaluated after log-transformation with a linear mixed model under consideration of sequence (2 level: AB, BA), period (2 level: visit 1 and visit 2) and product (2 level: placebo and verum) as fixed effects and participant as random effect. The linear mixed models were analyzed using SAS, version 9.4 (SAS Institute Inc., North Carolina, US). Differences between T_max_ were evaluated with non-transformed data using the cross-over analysis by strict separation of treatment effects from period effects. This was achieved via computing the treatment effects separately in two sequence groups formed via randomization according to Schumacher and Schulgen (27). The difference between treatment effects can be assessed by means of Wilcoxon rank sum test for independent samples using the intra-individual differences between the outcomes in both periods as the raw data. Unless declared otherwise, data of the clinical part are presented as mean ± 95% confidence interval (CI) using GraphPad Prism version 5.04, (GraphPad Software, Inc., San Diego, California, US).

## Results

### In vitro study

#### Effect of krill oil formulations on cell viability

The maximal applicable dose was determined using a cell viability assay (MTT conversion). Reduction of cell viability <75% of the control was considered cytotoxic. Based on the results of the viability assay digest concentration in medium was set to 2% digest solution of the respective formulation for transepithelial transport testing.

#### Effect of krill oil on silybin uptake

For the assessment of the influence of krill oil on the transepithelial transport of milk thistle extract, the concentration of silybin was measured in the basal compartment after a 24 h incubation period of Caco-2 insert models with a 2% digest solution of the respective formulation. Digestion solution without milk thistle extract served as control. As expected, silybin was not detectable in samples of the digest control.

**Fig. 1:**
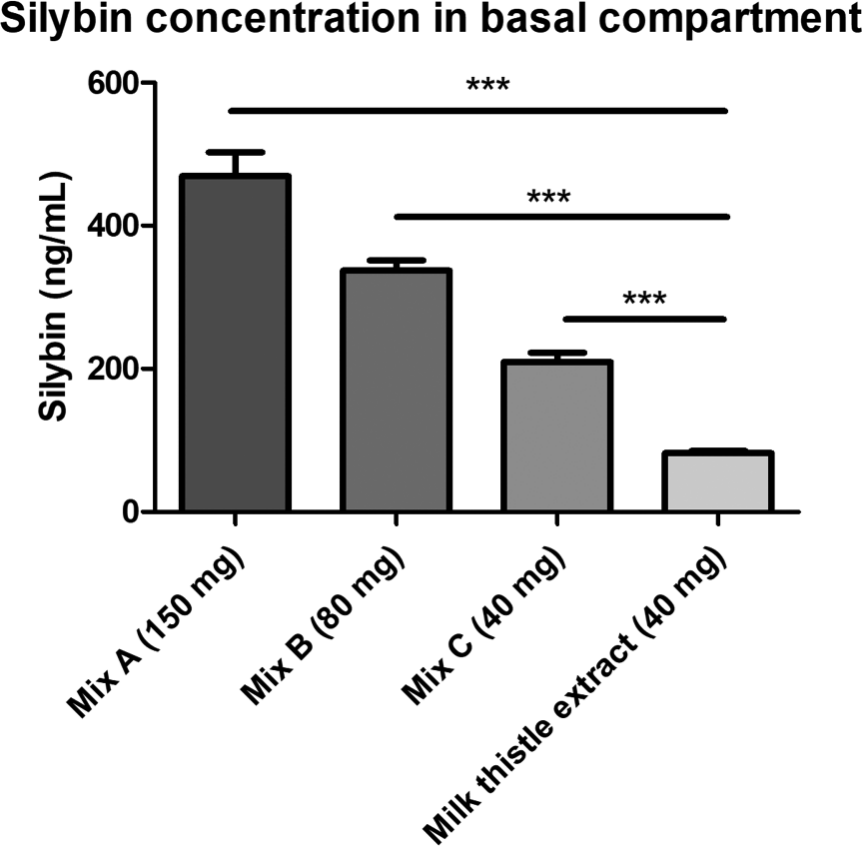
Transepithelial transport of silybin through Caco-2 models. Silybin concentration in basal compartment after 24 h incubation with the respective digest solution (2%). Values are given in ng/mL, mean ± SD, *n* = 6. Significance calculated with one-way ANOVA and Dunnett post test; all groups compared to milk thistle extract (40 mg) ***P < 0.001.

As seen in Fig. 1, there is a significant difference in transported silybin between the 40 mg milk thistle extract and the corresponding initial concentration of *Silybum marianum* extract in krill oil (40 mg). Silybin concentration in the basal compartment is increased by approximately 2.5-fold from 82.18 ± 3.4 ng/mL to 209.7 ± 12.3 ng/mL (*P* < 0.001), when delivered in krill oil formulation.

In addition, within the krill oil formulations, the silybin concentration in the basal compartment increased with increasing milk thistle extract concentration (209.7 ± 12.3 ng/mL vs. 337.2 ± 14.0 ng/mL vs. 469.2 ± 32.2 ng/mL [*P* < 0.001]).

Based on the data obtained, the following apparent permeability coefficient (P_app_) values for the permeation of silybin from the apical to basolateral compartment were calculated taking into account the dissolved portion after *in vitro* digest (25). The comparison between the three digestion solutions, with the same krill oil concentration and varying silymarin concentrations, shows that *P*_app_ values increased with increasing silymarin (see Table 1). Comparing the 40 mg milk thistle extract in krill oil with the corresponding milk thistle without krill oil reveals a significantly higher *P*_app_ value with 28% increase for the krill oil containing formulation (*P* < 0.001).

**Table 1:** *P*_app_ values (mean ± SD) of the different formulations

	P_app_ Silybin [x 10^-7^ cm/s]
Mix A (150 mg)	5.02 ± 0.34
Mix B (80 mg)	4.54 ± 0.19
Mix C (40 mg)	3.80 ± 0.22
Milk thistle extract (40 mg)	2.96 ± 0.12

When looking at the single silymarin components, a uniform transport pattern becomes apparent regardless of the concentration used (see Table 2). The dominant components in the apical supplementation solutions are silicristin (52.1%) and silybin (41.8%). Isosilibinin is only a minor component with 6.2% of total silymarin. In contrast, silybin is clearly the dominant component in the basal compartment with 79.4% of total silymarin. Silicristin, on the other hand, has a share of only 4.2% whereas the percentage of isosilibin increased to 16.4%. These results show that silybin is preferentially transported through the Caco-2 epithelium.

**Table 2:** Percentage of single silymarin components in the apical and basal compartments

	Percent of total Silymarin					
	Silicristin		Silybin		Isosilibin	
	apical	basal	apical	basal	apical	basal
Mix A (150 mg)	54.3	5.0	39.0	75.7	6.7	19.2
Mix B (80 mg)	49.5	3.7	44.1	80.3	6.3	16.0
Mix C (40 mg)	48.4	3.9	45.9	81.3	5.8	14.8
Milk thistle extract (40 mg)	56.1	4.2	38.0	80.1	5.9	15.7
Mean	**52.1**	**4.2**	**41.8**	**79.4**	**6.2**	**16.4**
SD	3.7	0.6	3.8	2.5	0.4	1.9

### In vivo study

#### Disposition of participants and baseline characteristics

**Fig. 2:**
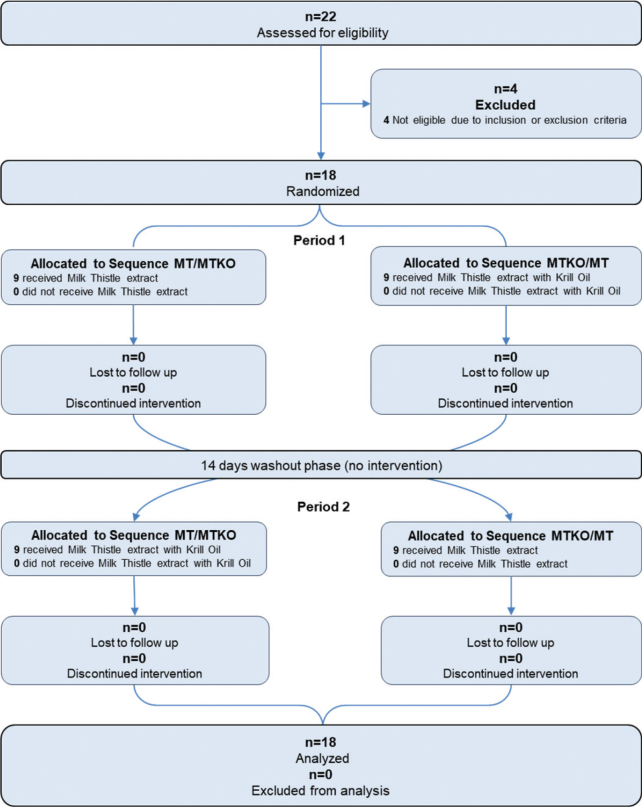
Disposition of participants.

Overall, 22 individuals were assessed for eligibility, of whom four did not meet the inclusion or exclusion criteria. Finally, 18 participants were randomized and completed the study with equal numbers of men and women participating (see Fig. 2).

Baseline characteristics of participants are displayed in Table 3. The participants of this study (*n* = 18) were overall healthy and non-smoking, had a mean age of 41.3 years (95% CI: 34.6–48.1) and an average BMI of 23.6 kg/m^2^ (95% CI: 22.2–24.9).

**Table 3:** Baseline characteristics (*n* = 18)

	Mean	95% CI
Age (years)	41.3	34.6–48.1
BMI (kg/m^2^)	23.6	22.2–24.9
Systolic blood pressure (mmHg)	127.2	123.5–130.9
Diastolic blood pressure (mmHg)	80.5	76.8–84.2
Heart rate (bpm)	60.2	56.1–64.2

Data are presented as mean and 95% CI.

#### Pharmacokinetic parameters

**Fig. 3:**
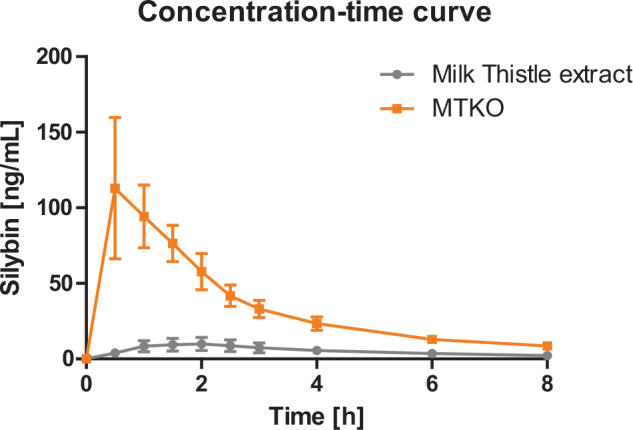
Concentration-time curve of silybin (ng/mL). Data presented as mean ± 95% CI.

After intake of the krill oil-based formulation and milk thistle extract a significant increase in silybin was determined for both products, though the effect was considerably more pronounced for the krill oil-based formulation (see Fig. 3).

**Fig. 4:**
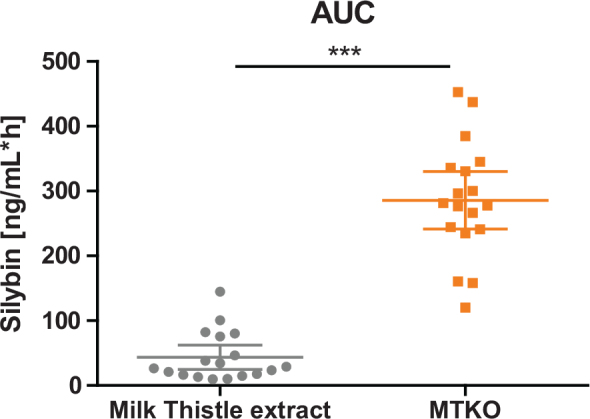
Distribution of AUC_0-8h_ of silybin [ng/mL*h]; Scatter diagram with mean ± 95% CI; *** *P* < 0.0001 (linear mixed model).

As illustrated in Fig. 4, the intake of the combined krill oil formulation resulted in a significantly higher (8.59-fold) AUC_0-8h_ of silybin compared to powdered milk thistle extract (*P* < 0.0001, see Table 4).

**Table 4:** Geometric mean ratio of silybin for AUC and C_max_

	Milk thistle extract LS mean (95% CI)	MTKO LS mean (95% CI)	Geometric mean ratio	*P*
AUC_0-8h_ [ng/mL*h]	31.6 (23.4–42.6)	271.2 (201.1–365.9)	8.59	<0.0001
C_max_ [ng/mL]	8.8 (6.3–12.2)	132.0 (94.9–183.6)	15.08	<0.0001

The maximum increase of silybin was significantly higher (>15-fold) for the combined krill oil formulation compared to powdered milk thistle extract (*P* < 0.0001, see Table 4). Despite the inter-individual variability in uptake efficacy, all participants showed a higher uptake of silybin from the combined krill oil formulation in comparison to the pure extract. Also, the time to reach C_max_ differed significantly between the formulations (*P* = 0.0016), with the maximum concentration of silybin after intake of the combined krill oil formulation being reached after 0.89 h (median 0.5 h) versus 1.56 h (median 1.5 h) after administration of milk thistle extract.

The utilization of sex (*P* = 0.7394) and BMI (*P* = 0.1306) as covariates did not demonstrate a significant impact on AUC_0-8h_. However, age was identified as a significant covariate in the linear mixed model for AUC_0-8h_ (*P* = 0.0459) with younger participants < 45 years (*n* = 9) showing a lower bioavailability than older participants ≥ 45 years (*n* = 9). However, the difference between age groups was only significant for powdered milk thistle extract (subgroup < 45 years: 22.14 ng/mL*h vs. 65.07 ng/mL*h for subgroup ≥ 45 years; unpaired t-test: *P* = 0.0109), but not for the combined krill oil formulation (subgroup < 45 years: 258.0 ng/mL*h vs. subgroup ≥ 45 years: AUC 313.6 ng/mL*h, *P* = 0.1945). The significant product effect (*P* < 0.0001) was seen across all models.

#### Safety assessment

Overall, four adverse events (headaches) were reported at the study days by four participants, with two occurring at the study site and additionally two in the evening of the study days after leaving the study site (1 x after milk thistle extract intake, 3 x after MTKO intake). Three of the headaches were judged as related to study procedure (e.g. prolonged sitting, early start in the morning and no coffee served during the day). None of the adverse events was judged as related to the study products. The findings of this study did not raise any safety concerns. No serious adverse events occurred during the study.

## Discussion

This study demonstrates that combining milk thistle extract with krill oil markedly enhances the bioavailability of silybin. Applying a sequential *in vitro* and *in vivo* design, our study first showed a 28% increase in the apparent permeability of silybin using a Caco-2 cell model. These *in vitro* findings were subsequently assessed and validated in a clinical study with healthy human participants. The krill oil formulation resulted in a statistically significant 8.59-fold and 15.08-fold increase in the AUC_0–8h_ and C_max_ of silybin, respectively, compared to the pure milk thistle extract administered under fasting conditions (*P* < 0.0001).

The new *in vitro* and human data extend current evidence that krill oil-based formulations can increase the bioavailability of silymarin. These results align with existing *in vitro* and *in vivo* evidence demonstrating that krill oil phospholipids can enhance the absorption of poorly water-soluble substances, such as curcumin (28, 29). Comparable to the use of krill oil, Gebhardt et al. showed that the use of Phosal®, a liquid formulation comprising PLs, phosphatidylcholine and medium-chain triglycerides, improved the transepithelial transport of curcuminoids in the Caco-2 cell model significantly compared to curcumin extract (30).

The substantial improvement in bioavailability is mechanistically plausible and attributable to the natural phospholipid content of krill oil (12). Phospholipids are amphipathic molecules that act as highly effective emulsifiers, forming micelles that can encapsulate lipophilic compounds like silybin (10, 13). This process enhances their solubilization in the aqueous environment of the gastrointestinal tract. In addition, phospholipids can modify the surface composition of fat droplets, promoting the binding of hydrolytic enzymes during digestion (31). In parallel, lipid-based formulations are known to stimulate the secretion of bile salts and lipases, which aids in the formation of mixed micelles and facilitates absorption (32). Beyond solubilization, bile salts lower intestinal surface tension to enhance transport (33), consistent with the increased silymarin bioavailability observed with bile salt–containing liposomes (34). These aspects are of particular importance given that the study products were administered under fasting conditions. A critical proposed mechanism is that absorption of the combined krill oil formulation may favor lymphatic transport, thereby bypassing hepatic first-pass metabolism, a major barrier for silybin’s systemic availability (35, 36). Notably, the use of natural, food-grade phospholipids from krill oil offers a toxicological advantage over synthetic surfactants, which can sometimes compromise membrane integrity or enzymatic activity (37, 38).

Our findings are consistent with a growing body of literature demonstrating the efficacy of phospholipid-based delivery systems. The observed 8.59-fold increase in AUC is comparable in magnitude to the 9.6-fold increase reported for a silybin-phosphatidylcholine complex (39) and aligns with other studies reporting significant, albeit variable, improvements (40, 41). However, between-study comparability is limited by substantial heterogeneity in dosing regimens, study populations, and study designs. The enhancing effect of krill oil is not limited to silybin; similar bioavailability improvements have been documented for other poorly soluble compounds, including curcumin and gingerols (29, 42). A recent study demonstrated that combining krill oil with ethyl esters significantly improved EPA and DHA bioavailability, with a 10.5-fold increase in iAUC_0-12h_ and a 4-fold increase in ∆C_max_ compared to ethyl esters alone (14). This underscores the potential of krill oil as a versatile platform for enhancing the delivery of various lipophilic bioactives.

Beyond the overall increase in exposure, the pharmacokinetic profile revealed a more rapid absorption of silybin from the krill oil formulation, with a median T_max_ of 0.5 h compared to 1.5 h for the pure milk thistle extract. This rapid onset is characteristic of efficient solubilization and uptake, and is consistent with observations from other phospholipid-based formulations (39, 41). Interestingly, our covariate analysis identified age as a significant factor, with older participants (≥45 years) exhibiting higher silybin bioavailability, particularly from the pure milk thistle extract. This may reflect age-related declines in hepatic first-pass metabolism (43) as silybin is subject to extensive hepatic biotransformation (44). The attenuation of this age effect in the krill oil group lends further support to the hypothesis that this formulation utilizes absorption pathways, such as lymphatic uptake, that partially circumvent hepatic first-pass elimination.

The clinical implications of this formulation extend beyond improved bioavailability. The combination creates a synergistic product containing bioactives that support both hepatic and cardiovascular health. Silymarin is well-documented for its antioxidant, anti-inflammatory, and anti-fibrotic properties in the liver (6). In MASLD silymarin has shown positive effects on different underlying pathophysiologic mechanism such as mitochondrial dysfunction and insulin resistance (6). Krill oil contributes highly bioavailable omega-3 PUFAs and choline, which are known to reduce liver fat and support cardiovascular function (15, 16). This multi-component approach presents a promising nutritional strategy for individuals at risk of MASLD, potentially serving as a valuable adjunct to primary lifestyle interventions.

Despite the new evidence this study has several limitations. The *in vitro* Caco-2 experiments did not include full particle characterization within the cell culture media; however, standardized procedures ensure the scientific validity of the direct comparison. The clinical study, while robust, was conducted in a small cohort of healthy individuals. Consequently, the extent to which these bioavailability findings can be generalized to populations at risk, including those with MASLD or compromised metabolic function, is uncertain; however, the observation that all 18 participants benefited from supplementation points to limited interindividual variation within individuals.

## Conclusions

This study demonstrates that formulating milk thistle extract with krill oil significantly enhances the oral bioavailability of its principal active constituent, silybin, in healthy human adults. This finding was supported by *in vitro* evidence from a Caco-2 cell model, which showed a marked increase in the transepithelial transport of silybin from the krill oil-based formulation.

These results suggest that the phospholipid-rich matrix of krill oil serves as an effective natural vehicle for improving the absorption of lipophilic bioactive compounds. Therefore, the examined approach may offer a promising strategy for enhancing the systemic exposure to silybin without the use of synthetic excipients. This innovative combination provides a matrix of nutrients – silybin, PUFAs, and choline – that could contribute to beneficial effects on both hepatic and cardiovascular health. While this study provides strong evidence for enhanced bioavailability following a single dose in healthy participants, future longer-term clinical studies are required to determine if this enhanced bioavailability translates into relevant health benefits.

## Supplementary Material


