# Supplementary material for: Enhanced bioavailability of a krill oil-based milk thistle extract formulation: in vitro and human studies
Source: Food Nutr Res. 2026 Jan 7;70:10.29219/fnr.v70.13256. doi: 10.29219/fnr.v70.13256 (PMC12829531; doi:10.29219/fnr.v70.13256)
Supplement: Supplementary file 1 [file FNR-70-13256-s1.pdf]

Supplementary Material

Enhanced bioavailability of a krill oil-based milk thistle extract formulation: *in vitro* and human studies

Supplementary Material 1: Listing of complete in- and exclusion criteria of clinical study

Inclusion criteria

(1) Healthy males or females (1:1)

(2) Age: 18 - 60 years

(3) BMI: 19 to 30 kg/m^2

(4) Current Non-smoker

(5) Participant is able and willing to sign the Informed Consent Form prior to screening evaluations

(6) Participant is in good physical and mental health as established by medical history, vital signs, results of biochemistry, hematology

(7) If applicable, stable intake of chronic medication of at least 4 weeks

Exclusion criteria

(1) Relevant history or presence of any severe medical disorder, potentially interfering with this study (e.g. mal absorption, chronic gastro-intestinal diseases (colitis ulcerosa, Crohn's IBS, peptic ulcers, celiac disease), heavy depression, diabetes, heavy liver disease, immunodeficiency, pancreas insufficiency, acute cancers within last 3 years except basal cell carcinoma of the skin, etc.)

(2) A significant CVD event within last 3 mo. incl. myocardial infarction, stroke, congestive heart failure

(3) Regular intake of drugs or supplements possibly interfering with this study (e.g. milk thistle supplements, krill oil or fish oil products etc.) within 2 weeks prior to study start or during study

(4) Chronic intake of substances affecting blood coagulation (e.g. acetylic acid, anticoagulants (e.g. Marcumar), diuretics, thiazides), which in the investigator's opinion would impact participant safety

(5) General Safety & Laboratory exclusion criteria: Patients will be excluded from the study based on the following criteria:

- Hemoglobin: < 11.0 g/dL (women); < 12.0 g/dL (men);
- AST or ALT >3 x Upper Limit of Normal

(6) Blood donation within 1 month prior to study start or during study

(7) Participants with history of drug, alcohol or other substance abuse, or other factors limiting their ability to co-operate during the study

(8) Pregnancy, breast feeding or intention to become pregnant during the study

(9) Participation in another clinical study within the last 4 weeks and concurrent participation in another clinical study

(10) A known allergy or hypersensitivity to any of the ingredients of the study products e.g. crustaceans and Asteraceae

(11) Participants considered inappropriate for the study by investigators, including participants who are unable or unwilling to show compliance with the protocol

Supplementary Material 2: Technical Annex — LC–MS/MS Method Silybin

1. Instrumentation

LC system: Waters Acquity H-Class UPLC

Mass spectrometer: Waters Xevo TQS-µ

Detector: Waters eλ Diode Array Detector

Column manager: Waters Acquity CM, 30 °C

Autosampler: Waters Acquity FTN

Column: Acquity UPLC BEH C18, 1.7 µm

2. LC Conditions

Flow rate: 0.30 mL/min

Injection volume: 1 µL

Column temperature: 30 °C

Mobile phases

A: Water + 0.1% formic acid

B: Acetonitrile + 0.1% formic acid

C: Methanol (not used)

D: Isopropanol (not used)

Gradient

Time (min)	Flow (mL/min)	%A	%B	%C	%D
0.00	0.300	75	25	0	0
3.00	0.300	25	75	0	0
6.00	0.300	25	75	0	0
6.60	0.300	75	25	0	0
9.00	0.300	75	25	0	0

Mass Spectrometry Conditions

Ionization mode: ESI negative

Functions acquired:

- **Function 1:** MRM
- **Function 2:** MS/MS full scan
- **Function 3:** PDA (UV)

Source and Gas Settings (Function 1 & 2)

Parameter	Value
Capillary voltage	2.21 kV
Cone voltage (global)	20 V
Source temperature	150 °C
Desolvation temperature	550 °C
Cone gas flow	20 L/h
Desolvation gas flow	850 L/h

Table S1: MRM settings of the final ESI+-MS/MS method.

Analyte	Parent ion (m/z)	Daughter ion (m/z)	Cone voltage [V]	Collision energy [V]	Ion transition type
**Silybin**	**481**	**125**	**58**	**26**	**Q**
**Silybin**	**481**	**152**	**55**	**38**	**q**
**Silybin**	**481**	**301**	**31**	**16**	**q**
**Naringenin**	**271**	**151**	**20**	**18**	**Q**
**Naringenin**	**271**	**119**	**20**	**24**	**q**

Q: Quantifier ion transition, q: qualifier ion transition
